# Functional analyses reveal an important role for tyrosine residues in the staphylococcal multidrug efflux protein QacA

**DOI:** 10.1186/1471-2180-8-147

**Published:** 2008-09-16

**Authors:** Jingqin Wu, Karl A Hassan, Ronald A Skurray, Melissa H Brown

**Affiliations:** 1 School of Biological Sciences, The University of Sydney, Sydney, New South Wales, 2006, Australia; 2 School of Biological Sciences, Flinders University, Adelaide, South Australia, 5042, Australia; 3 Retroviral Genetics Laboratory, Westmead Millennium Institute, University of Sydney, New South Wales, Australia; 4 Department of Chemistry and Biomolecular Sciences, Macquarie University, New South Wales, Australia

## Abstract

**Background:**

The staphylococcal QacA multidrug efflux protein confers resistance to an exceptional number of structurally unrelated antimicrobial compounds. Aromatic amino acid residues have been shown to be highly important for the transport function of several multidrug transporters and are intimately involved in multidrug binding. This study investigated the structural and functional importance of the seven tyrosine residues in QacA by examining the phenotypic effect of incorporating conservative (aromatic) and non-conservative (non-aromatic) substitutions for these residues.

**Results:**

Determination of the resistance profiles and analysis of drug transport assays revealed that non-conservative substitutions for most tyrosine residues influenced the QacA drug recognition spectrum. However, an aromatic residue at three tyrosine positions, 63, 410 and 429, was of importance for QacA-mediated transport and resistance to the majority of substrates tested.

**Conclusion:**

A tyrosine or phenylalanine residue at amino acid positions corresponding to 63 of QacA in related drug efflux proteins is found to be highly conserved. Therefore, an aromatic side chain at this position is likely to partake in a function common to these drug transporters, such as proton translocation or essential intramolecular contacts, whereas aromatic residues at the non-conserved 410 and 429 positions are expected to mediate a QacA-specific function, possibly forming or stabilising part of the QacA drug binding region.

## Background

The QacA multidrug efflux protein confers resistance to at least 30 structurally distinct monovalent or bivalent cationic lipophilic antimicrobials from at least 12 different chemical families [1,2]. Furthermore, genes encoding *qacA *or a highly related derivative are carried by widespread clinical isolates of the human pathogen *Staphylococcus aureus*[3-5]. Therefore, the QacA multidrug resistance protein facilitates an efflux mechanism by which strains of *S. aureus *may currently overcome control measures, such as the use of biocides within the clinical environment, and spread to new hosts [1,3].

**Figure 1:**
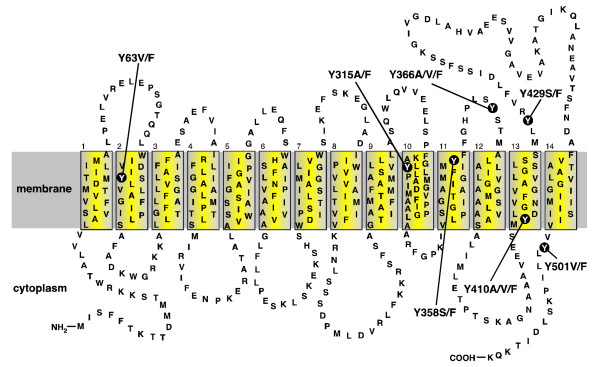
**Secondary structure model of the QacA multidrug transport protein**. Topology of the QacA multidrug efflux protein based on hydropathy predictions and solvent accessibility analyses [6,7,10]. The membrane is depicted as a grey shaded band and the 14 TMS of QacA are shown in boxes numbered 1–14. The locations of the seven tyrosine residues and their amino acid substitutions within QacA are shown.

The QacA polypeptide is composed of 514 amino acid residues, organised into 14 α-helical transmembrane segments (TMS) (Figure 1) [6,7] and is classified as a member of the drug:H^+ ^antiporter (DHA) 2 family of the major facilitator superfamily (MFS) of transport proteins [8]. Drug efflux mediated by QacA is powered by the proton-motive-force and can be described using Michaelis-Menton kinetics [9]. The drug binding region within QacA appears to contain distinct binding sites for monovalent and bivalent cationic antimicrobials [9]. Nonetheless, biochemical studies of TMS lining the binding region have suggested that these binding sites may be in proximity [10]. A clear requirement for an acidic residue in the recognition of bivalent cationic substrates by QacA has been established [6,10,11]. However, the location of this residue within the binding region is flexible and appears to influence the bivalent drug recognition spectrum of the QacA protein [11].

Multidrug binding regions fundamentally differ from binding sites required to bind only a single drug or group of closely related drugs, in that they accommodate and form stabilising interactions with large numbers structurally diverse compounds. A number of studies have attempted to delineate the mechanisms by which multidrug binding occurs [12]. These studies, including the determination of several drug-bound crystal structures for multidrug binding proteins [13-16], have demonstrated that multidrug binding may be described as a process of "induced-fit", i.e., each compound localises to a position or positions within a multidrug binding region where it is best accommodated and its binding is stabilised by a particular sub-set of amino acid side chains lining the region. Common among the sub-sets of residues that mediate interactions with drug compounds are aromatic residues [13-16]. This is not surprising since the benzene group of aromatic side chains potentiates a range of molecular interactions which could stabilise binding of the typically hydrophobic, planar and often cationic substrates of multidrug binding proteins, including stacking, hydrophobic, π-π and cation-π interactions [17]. Beyond drug binding within a secondary multidrug transport protein such as QacA, these types of molecular interactions could also play a role in other functions, such as translocation of coupling ions or the mediation of stabilising intramolecular contacts [18,19].

A previous study investigating the significance of tryptophan residues within the QacA transport protein demonstrated that those more highly conserved among QacA-related transporters were the most functionally significant [20]. In the current study the importance of the benzene moiety within the tyrosine residues of the QacA transport protein was determined. Of the seven tyrosine residues in the QacA polypeptide, four are known or predicted to be membrane embedded and three are thought to be located within loop regions (Figure 1) [6,7,10]. The aromaticity of the tyrosine residue at position 63 of QacA (Y63) is highly conserved among DHA family transporters; a tyrosine, or in some cases a phenylalanine, is almost exclusively found at this position among these proteins [8]. Therefore, this residue may function in a role common to these drug efflux proteins, whereas the remaining six tyrosine residues could partake in functions unique to QacA. Conservative and non-conservative substitutions in terms of side-chain aromaticity, phenylalanine and alanine, serine and/or valine, respectively, were made for each of the tyrosine residues in QacA and the mutants functionally analysed (Figure 1).

## Methods

### Strains, plasmids, media and reagents

The *E. coli *strain DH5α [*supE*44 Δ*lac*U169 (ϕ80*lac*ZΔM15) *hsdR*17 *recA*1 *endA*1 *gyrA*96 *thi*-1*relA*1] [21] was used for routine plasmid cloning procedures, Western blot analyses and ethidium transport assays. *E. coli *BHB2600 (*supE*, *supF*, *hsdR*, *met*) [22] was used in MIC analyses. The plasmids used were pBluescript II SK (Stratagene) and the pBluescript-based *qacA *clone, pSK4322 [10]. Cultures of *E. coli *cells were grown in Luria-Bertani media. Ampicillin was used at a concentration of 100 μg ml^-1 ^for plasmid selection where appropriate.

Benzalkonium chloride, chlorhexidine dihydrochloride, dequalinium chloride, ethidium bromide, pyronin Y and carbonyl cyanide *m*-chlorophenylhydrazone were purchased from Sigma and diamidinodiphenylamine dihydrochloride was obtained from Rhone-Poulenc Rorer (Dagenham, UK). All other chemicals were of at least reagent grade and purchased from commercial sources.

### Substitution of wild-type tyrosine residues

The tyrosine residues within QacA were individually substituted using site-directed mutagenesis by the Stratagene QuikChange method. Pairs of complementary oligonucleotide primers (GeneWorks, Australia) were designed with two positions of degeneracy to insert alanine, phenylalanine, serine or valine substitutions for each of the seven tyrosine codons in the *qacA *gene carried on pSK4322. The primer pairs also incorporated a silent mutation which introduced an endonuclease restriction site within the *qacA *sequence. The *qacA *gene sequences within mutagenised pSK4322 derivatives, containing an introduced restriction site, were sequenced (Australian Genomic Research Facility, Brisbane) in their entirety to identify the incorporated amino acid changes and to ensure that undesired secondary mutations had not been introduced elsewhere in the genes. Gene sequences were stored and analysed using Sequencher version 4.2.2 (Gene Codes Corp.).

### Western blot analyses

*E. coli *DH5α cells harbouring pSK4322-derived plasmids expressing QacA mutants were cultured to OD_600 _= 0.6. Equal volumes of cells were collected, lysed and electrophoresed on a 10% sodium dodecyl sulphate polyacrylamide gel electrophoresis gel. Proteins were transferred to polyvinylidene difluoride membrane and probed using an anti-QacA polyclonal antiserum [10] and a colourimetric detection system. Membranes were scanned with a BioRad GS-710 calibrated imaging densitometer and analysed by Quantity One^® ^software (BioRad). Mutant protein expression levels were expressed as a percentage of the wild-type protein.

### Minimum inhibitory concentration analyses

MIC analyses were conducted using solid Luria-Bertani agar media supplemented with antimicrobial compounds in the following concentration ranges: benzalkonium, 20–70 μg ml^-1^, chlorhexidine 1–20 μg ml^-1^, dequalinium 20–400 μg ml^-1^, diamidinodiphenylamine 50–500 μg ml^-1^, ethidium 50–1800 μg ml^-1^, and pyronin Y 25–600 μg ml^-1^. *E. coli *BHB2600 cells carrying pSK4322-derived plasmids encoding each QacA mutant protein were grown to equivalent densities in the late exponential phase then diluted 1:200 and replica plated onto plates containing increasing concentrations of antimicrobials. Plates were incubated at 37°C for up to 48 hours and the MIC determined as the lowest concentration of antimicrobial compound required to fully inhibit bacterial growth.

### Kinetic transport studies

Fluorimetric ethidium transport studies were conducted essentially as described previously [9,23]. Briefly, *E. coli *DH5α cells carrying pSK4322-derived plasmids encoding each QacA mutant protein were grown to OD_650 _= 0.6, then washed and resuspended in 20 mM HEPES buffer. Cells were loaded with ethidium at concentrations ranging from 0.1–25 μM in the presence of 10 μM carbonyl cyanide *m*-chlorophenylhydrazone. Ethidium loaded cells were again washed and resuspended in 20 mM HEPES, before transport was initiated by the addition of 500 mM sodium formate. Transport was monitored fluorimetrically using a Hitachi 4500 fluorescence spectrophotometer with excitation and emission wavelengths of 530 and 610 nm, respectively.

## Results

### Construction of site-directed QacA tyrosine mutants

Site-directed mutagenesis using oligonucleotide primers degenerate at two nucleotide positions within each targeted codon was used to exchange tyrosine codons within the *qacA *gene carried on pSK4322 for alanine, valine, serine, and/or phenylalanine. Phenylalanine substitutions for each tyrosine residue were specifically isolated to determine the effect of conservative (aromatic) tyrosine substitutions on QacA function. Additionally, mutants encoding at least one of alanine, serine or valine in place of each tyrosine residue were isolated to examine the importance of side-chain aromaticity at tyrosine positions for the catalytic activity of the QacA transporter. In total, genes encoding 16 tyrosine-substituted QacA mutants were isolated for functional analyses (Figure 1). The expression of these QacA variants was assessed using semi-quantitative Western blot analysis. Each of the QacA mutants was shown to be expressed at a level facilitating functional studies, ranging from 32 to 132% of wild-type QacA (Table 1).

**Table 1:** Phenotypic profiles of QacA tyrosine mutants

QacA mutation	QacA expression (% wt QacA)^c^	minimum inhibitory concentration (μg ml^-1^)^a.b^					
		monovalent substrates			bivalent substrates		
			
		dyes		Qacs^d^		Bg	Di
					
		Et	PyY	Bc	Dc	Ch	Dd
wt QacA	100	1200	500	50	350	12	300
vector	N/A	400	50	30	100	2	100
Y63V	86	200	50	30	150	4	100
Y63F	50	1400	400	50	250	12	300
Y315A	64	800	500	50	300	12	400
Y315F	75	1000	500	50	300	12	300
Y358S	80	600	400	50	100	8	200
Y358F	34	1400	400	50	300	16	300
Y366A	106	1200	300	50	100	12	300
Y366V	50	1200	400	50	300	12	300
Y366F	99	800	500	50	300	12	400
Y410A	132	400	50	45	100	3	100
Y410V	67	200	50	30	100	7	300
Y410F	58	200	100	50	250	12	300
Y429S	57	200	200	30	250	4	100
Y429F	32	1400	500	60	300	14	400
Y501V	36	800	500	50	300	12	300
Y501F	47	1200	500	50	350	12	400

^a ^MIC values were determined in *E. coli *BHB2600 cells expressing wild-type QacA or a mutant derivative and represent the lowest concentration of substrate required to fully inhibit bacterial growth.

^b ^MIC experiments were conducted in at least triplicate and the median value is presented.

^c ^QacA protein expression levels are the average of two repeat experiments.

^d ^Abbreviations: Qacs, quaternary ammonium compounds; Bg, biguanidines; Di, diamidines; Et, ethidium; PyY, pyronin Y; Bc, benzalkonium; Dc, dequalinium; Ch, chlorhexidine; Dd, diamidinodiphenylamine.

### Drug resistance profiles of the QacA tyrosine mutants

In order to gauge the functional consequences of QacA tyrosine substitutions in the recognition and transport of a range of chemically diverse QacA substrates, minimum inhibitory concentration (MIC) analyses were conducted. The resistance levels conferred by the QacA mutant proteins to six compounds, representing five different chemical classes were tested: monovalent dyes (ethidium and pyronin Y); monovalent quaternary ammonium compounds (benzalkonium); bivalent quaternary ammonium compounds (dequalinium); biguanidines (chlorhexidine); and diamidines (diamidinodiphenylamine).

Phenylalanine substitutions for the majority of the tyrosine residues in QacA did not have a significant bearing on the capacity of QacA to mediate drug resistance (Table 1). Indeed, only the Y410F derivative was notably affected and then only in its capacity to mediate resistance to the monovalent dyes ethidium and pyronin Y. Similarly, the non-aromatic substituted Qac mutants, Y315A, Y366V and Y501V, had no significant effect on drug resistance. In contrast, Y63V, Y410A, Y410V, and Y429S non-aromatic substitutions significantly reduced or abolished QacA-mediated resistance to most representative QacA substrates. Thus, these results suggest that aromatic residues at positions 63, 410 and 429 participate in an aspect of QacA-mediated transport common to all substrates. These aromatic residues may function mechanistically, e.g., in proton translocation or in facilitating intramolecular conformational transitions required for substrate transport. Alternatively, these residues could compose part of the QacA binding region universal to all substrates or stabilise the binding region in the absence of substrate. Of the remaining amino acid substitutions examined, three had slight differential effects on substrate resistance where Y358S and Y366A abolished QacA-mediated resistance to the bivalent quaternary ammonium compound dequalinium.

### Kinetics of ethidium transport mediated by QacA tyrosine mutants

**Figure 2:**
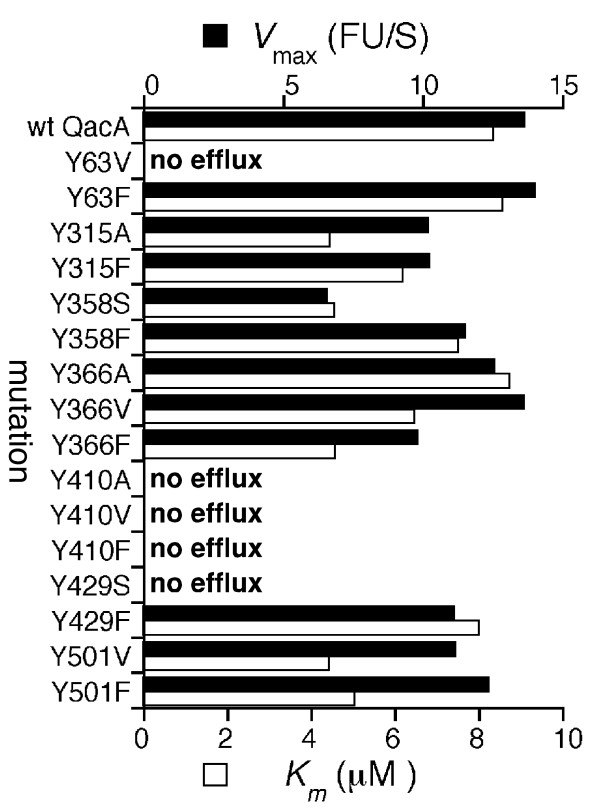
**Kinetics of ethidium transport by QacA tyrosine mutants**. Cells expressing each QacA derivative were loaded with various concentrations of ethidium and efflux monitored fluorimetrically after energisation. *V*_max _values (black bars) represent the maximal change in fluorescent units per second (ΔFU S^-1^) and *K*_*m *_values (white bars) are presented as μM ethidium. Transport assays were conducted in at least triplicate and the results of a representative experiment are shown.

To complement the MIC analyses the kinetics of ethidium transport were determined for each tyrosine-substituted QacA mutant. In line with their inability to confer resistance to ethidium, the Y63V, Y410A/V/F and Y429S QacA mutants failed to mediate ethidium efflux (Figure 2). Furthermore, mutants conferring only low-level ethidium resistance, such as Y358S, displayed lower maximal rates of transport than the wild-type protein and other functional mutants (Figure 2). The remaining mutants displayed rates of ethidium transport approximately in line with the wild-type QacA protein. The *K*_*m *_values derived for the mutants functional in ethidium transport were approximately equal to, or in some cases less than, that determined for the wild-type QacA protein and do not point towards reduced ethidium recognition as a result of the incorporated mutations (Figure 2).

## Discussion

The benzene moiety of aromatic amino acids affords these residues versatility in molecular interactions, which is ideal within the context of a multidrug transporter. For example, drug binding, a key step in drug translocation by multidrug transporters, requires the use of multidrug binding regions that accommodate and stabilise the binding of a diverse array of chemical compounds. Such binding regions benefit from side-chains, such as those of aromatic residues, capable of facilitating a range of molecular contacts with the various structural moieties composing different drug substrates. Indeed, multidrug binding regions, including those within the *Escherichia coli *multidrug transporters AcrB and EmrE and the *S. aureus *QacA regulator protein QacR, which binds a similar range of lipophilic cationic substrates to the QacA transporter, are lined by numerous aromatic residues [13-16,24]. A number of these residues have been determined to facilitate diverse structural interactions with different substrates, making them key mediators of multidrug binding. Furthermore, aromatic residues, Y92 and Y93 in particular, were seen to function as "drug surrogates" that stabilise the multidrug binding region of the QacR regulator in the absence of drug and are expelled upon drug binding [16]. Interestingly, the expulsion of Y92 and Y93 in QacR leads to a conformational change that dissociates the protein from DNA and allows *qacA *transcription [16]. It is interesting to speculate that a similar scenario within a multidrug transporter could provide the impetus for initiating a translocation cycle. Beyond their roles within multidrug binding regions, aromatic residues have been shown to influence proton coupling within multidrug transporters and may also play important structural roles in mediating TMS interactions or in guiding the depth of TMS membrane insertion [18,25,26].

This study investigated the importance of a benzene moiety at amino acid positions occupied by tyrosine residues with the QacA multidrug transporter (Figure 1). Overall, the tyrosine residues in the QacA protein were readily replaceable with phenylalanine, indicating that the hydroxyl group of the tyrosine side chains is not essential in QacA-mediated transport function, although, a hydroxyl moiety may be important at position 410 to allow recognition of the monovalent dyes ethidium and pyronin Y. In contrast, the benzene groups of the QacA tyrosine residues were less dispensable. This was particularly evident for the QacA Y63, Y410 and Y429 tyrosines, which when replaced with non-aromatic residues resulted in large reductions or the abolition of resistance to most representative compounds and the complete loss of ethidium transport capacity (Table 1; Figure 2). This suggests that aromatic side chains at these positions are of general importance for QacA-mediated transport function. Since the aromaticity of Y63 in QacA is highly conserved among DHA family transporters with varied drug recognition profiles [8,23], it is likely that the benzene moiety of this side chain functions in a mechanistic role common to these proteins, possibly proton coupling as demonstrated for Y4 of the *E. coli *EmrE transporter [18]. Although Y410 and Y429 of QacA are not conserved among related proteins, these residues may also function mechanistically in the QacA transport process, possibly in mediating contacts between distal parts of the QacA polypeptide. Given that the Y410F substitution affected only the transport of monovalent dyes, Y410 may face into the QacA multidrug binding region and mediate interactions with these substrates via its hydroxyl group. In such a location, this residue may also interact with other substrates via its benzene moiety or could function similarly to Y92 and Y93 of QacR and stabilise the QacA binding region in the absence of drugs. Non-aromatic substitutions for Y358 and Y366 prevented resistance to dequalinium, implying that these residues may function in dequalinium binding or stabilise the dequalinium binding site.

## Conclusion

Overall, the results of this study suggest a functional need to maintain the aromaticity of a number of tyrosine residues within the QacA transport protein. In several cases, modification of these residues for a non-aromatic residue resulted in an altered drug recognition spectrum, hinting at an involvement of these side chains in drug binding. This finding is in keeping with previous studies of multidrug binding proteins which have identified aromatic residues as key mediators of multidrug binding.

## Abbreviations

DHA: drug:H^+ ^antiporter; MFS: major facilitator superfamily; MIC: minimum inhibitory concentration; TMS: α-helical transmembrane segment.

## Authors' contributions

JW performed the molecular experiments. KAH performed the protein analyses and drafted the manuscript. RAS and MHB conceived the study, and participated in its design and coordination and helped to draft the manuscript. All authors read and approved the final manuscript.
